# Nanobiosensors Design Using 2D Materials: Implementation in Infectious and Fatal Disease Diagnosis

**DOI:** 10.3390/bios13020166

**Published:** 2023-01-20

**Authors:** Nandita Singh, Daphika S. Dkhar, Pranjal Chandra, Uday Pratap Azad

**Affiliations:** 1 Department of Chemistry, Guru Ghasidas Vishwavidyalaya, Bilaspur 495009, CG, India; 2 Laboratory of Bio-Physio Sensors and Nanobioengineering, School of Biochemical Engineering, Indian Institute of Technology (BHU), Varanasi 221005, UP, India

**Keywords:** nanobiosensors, immunosensors, diagnosis, point of care, nanomaterials, nanobiodevices, two dimensional nanomaterials

## Abstract

Nanobiosensors are devices that utilize a very small probe and any form of electrical, optical, or magnetic technology to detect and analyze a biochemical or biological process. With an increasing population today, nanobiosensors have become the broadly used electroanalytical tools for the timely detection of many infectious (dengue, hepatitis, tuberculosis, leukemia, etc.) and other fatal diseases, such as prostate cancer, breast cancer, etc., at their early stage. Compared to classical or traditional analytical methods, nanobiosensors have significant benefits, including low detection limit, high selectivity and sensitivity, shorter analysis duration, easier portability, biocompatibility, and ease of miniaturization for on-site monitoring. Very similar to biosensors, nanobiosensors can also be classified in numerous ways, either depending on biological molecules, such as enzymes, antibodies, and aptamer, or by working principles, such as optical and electrochemical. Various nanobiosensors, such as cyclic voltametric, amperometric, impedimetric, etc., have been discussed for the timely monitoring of the infectious and fatal diseases at their early stage. Nanobiosensors performance and efficiency can be enhanced by using a variety of engineered nanostructures, which include nanotubes, nanoparticles, nanopores, self-adhesive monolayers, nanowires, and nanocomposites. Here, this mini review recaps the application of two-dimensional (2D) materials, especially graphitic carbon nitride (g-C_3_N_4_), graphene oxide, black phosphorous, and MXenes, for the construction of the nanobiosensors and their application for the diagnosis of various infectious diseases at very early stage.

## 1. Introduction

**Figure 1:**
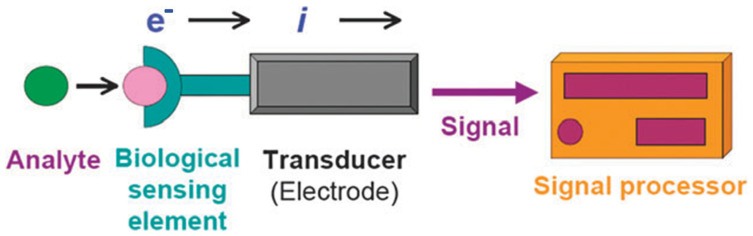
A schematic showing a nanobisensors with electrochemical transducer and other components. Reproduced with permission from [3].

Biosensors are devices designed to employ a biological recognition element, for example antibodies, enzymes, or oligonucleotides, and convert the biochemical response resulting from the interaction of this element with the analyte of interest into a quantifiable physical signal and allow for the detection of the same at extremely low concentrations [1,2,3,4,5,6]. Transducer, bioreceptor, and detector are the three fundamental parts of a biosensor used to identify various main metabolites, immunological compounds, and other substances. The popularity of affinity-based biosensors stems from the fact that they can detect changes at a contained surface, making sensing targets in solution easier. They achieve this by initially employing an immobilized capture probe, which in turn binds with the target or analyte of interest in a selective manner. Electrochemical biosensors combine the intrinsic bio-selectivity of the biological component with the sensitivity of electroanalytical techniques. The biological component of the nanobiosensor (Figure 1) detects its analyte, leading to a binding or catalytic event that eventually generates an electrical signal measured by a transducer that is proportionate to the concentration of the analyte [7,8,9,10,11,12,13,14,15,16,17,18].

**Figure 2:**
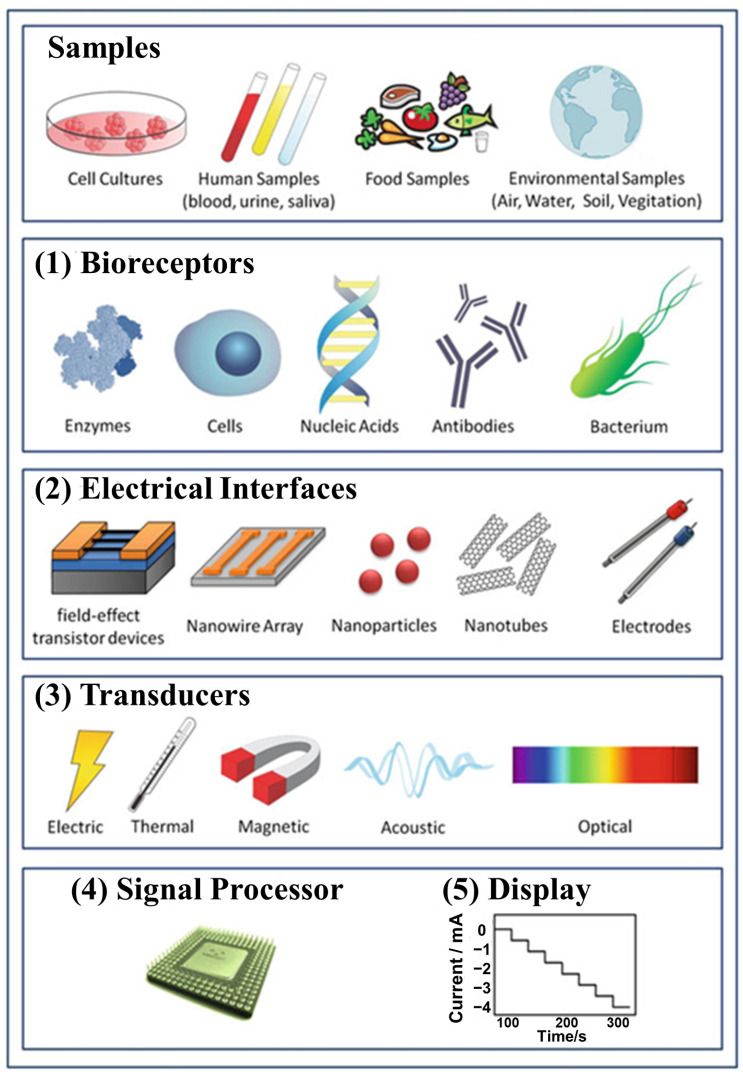
Various components of a typical nanobiosensor. Reproduced with permission from [22].

A nanobiosensor is a biosensor, which is of nanoscale size. Any nanoscale device with features, such as quantifying and specifically detecting a relatively low amount of a substance or even a single particle of interest, is referred to as a nanobiosensor, or in other words, “nanosensors are chemical or physical sensors made from nanoscale components (often microscopic or submicroscopic in size)” [19,20]. The field of nanobiosensors combines several academic fields, including material science, physics, chemistry, biochemistry, and engineering [21,22]. The distinct physicochemical characteristics of nanomaterials (NMs) show promise for achieving a high sensitivity, precision, and dependability of nanobiosensors, particularly for medical-related applications, such as monitoring and detecting the onset of a disease. It is made up of a signal transducer and recognition components. The usual components of a biosensor are shown in Figure 2. A biological recognition molecule is frequently mounted on the surface of a signal transducer in nanobiosensors. Due to the heterogeneous nature of the interaction between the biorecognition element and the target, the performance of the nanobiosensor is dependent on the construction and fabrication of the biosensing interface [23]. For the molecular detection of biomarkers linked to illness diagnosis, nanobiosensors are frequently used. The use of novel nanomaterials in biosensors has a considerable impact on that field of study. Higher sensitivity and faster reaction times have been achieved using nanobiosensors by using large surface area nanomaterials [24]. This article outlines the developments in illness diagnosis, especially through the use of nanobiosensors to detect molecular biomarkers, such as proteins and nucleic acids.

Numerous synthetic compounds, such as antibodies, nucleic acids, enzymes, aptamers (which are either oligonucleotide or peptide molecules), and others, are examples of recognition elements [19]. Medical diagnostics, in particular, use nanoparticles with excellent properties for binding nucleic acid or peptide chemistry, thereby increasing detection sensitivity. Recent advances in nanobiosensor technology made it simple to use them for clinical diagnostics and biochemical analyses [25,26,27,28,29,30]. The three most well-known methods for obtaining materials at the nanoscale are bottom-up assembly, top-down lithography, and molecular self-assembly. So far, several reviews and studies focusing on the advancement of biosensors based on nanomaterials have been published. In this review, we have tried to cover various types of nanomaterials, mainly two-dimensional (2D) materials, such as graphitic carbon nitride (g-C_3_N_4_), graphene oxide, black phosphorous, and MXenes, which has been used for the construction of electrochemical nanobiosensors, which have been employed for the detection and diagnosis of infectious diseases. As compared to other materials, 2D materials are exceptionally effective in interacting with outside stimuli due to their inherent openness and for device control; it is easier to mechanically, electronically, optically, and magnetically modify the material properties in 2D systems than in 3D bulk and other systems [31].

### 1.1. Principle and Operation of Nanobiosensors

Nanobiosensors are tools that use a small probe and any electrical, optical, or magnetic technology to measure a biochemical or a biological event. Nanobiosensors are a new class of biosensors made possible by the intersection of current breakthroughs in nanotechnology and sophisticated manufacturing technology in electronics. This has ushered in a new era of bionanotechnology for disease diagnosis. A nanobiosensor can be used to identify biological target agents, such antibodies, infections, nucleic acids, and metabolites [32].

The basic idea is to bind relevant bioanalytes to bioreceptors, which then modulate the physiochemical signal related to the binding. The physiochemical signal is then captured and transformed into an electrical signal by a transducer. It is kept track of when a signal changes in terms of its electric potential, current, conductance, impedance, electromagnetic radiation’s intensity and phase, mass, temperature, and viscosity. The absence or presence of bioagents is quantified by assessing the variations in some or all of these parameters. The transducer is paired with nanomaterials to create a biosensor, and the nanostructures in nanobiosensors serve as an intermediary layer between biological agents and physicochemical detector elements or biological agents. The biological layer is the site for precise contact with the target of interest, and this interaction is transformed into a quantifiable effect by the transducer [33,34,35]. For example, the analyte–bioreceptor interaction is typically translated by mechanical transducers into a shift in resonant frequency or bending; optical transducers typically translate this phenomenon into a shift in intensity or light frequency, and electrochemical transducers typically translate this phenomenon into a shift in current, potential. The reading system ultimately determines how much each change has altered. The reading system rates the encounter’s physical consequences [36,37]. A suitable reading device may detect physical events, such as alterations in bending, variations in resonant frequency, fluctuations in electrochemical outputs, such as potential or current, and variations in optical properties.

### 1.2. Desirable Properties of Nanobiosensor

There are main features that an ideal nanobiosensor should have for precise and sensitive determinations. Linearity must be wide enough to detect high analyte concentrations. It should be sufficiently sensitive depending on the analyte concentration exhibiting high selectivity to obtain reliable results. The time to achieve 95% of the total response should be as short as possible. Properties, such as biocompatibility, stability at usual storage circumstances, and stability, also contribute to the high specificity of nanobiosensors toward the analyte. Nanobiosensors must be distinct and unrestrained of any physical factors, such as agitation, pH, etc. In addition, the nanobiosensor designed as a disposable sensing platform is another important feature that attracts users for on-site analysis.

Here, the 2D material-based nanobiosensors, especially graphitic carbon nitride (g-C_3_N_4_), graphene, black phosphorous, and and MXenes, will be discussed one by one, which has been used in the construction of nanobiosensors, which were employed for the diagnosis of various infectious and fatal diseases. 

### 1.3. Graphitic Carbon Nitride-Based Nanobiosensors

Graphitic carbon nitride (g-C_3_N_4_), over the years, gained wide attention owing to its lack of metal, non-toxicity, ease of manufacture, low cost, and acceptable band gap in the visible spectrum region. It is commendably stable under ambient circumstances and also shares a structure with graphite [38]. It has emerged as a potential material in photocatalysis, fluorescence and gas sensors, field emitters, electrodes for fuel cells, and hydrogen storage due to its numerous nitrogen active sites and excellent catalytic activity [39,40]. It is significant to highlight that synthesized bulk g-C_3_N_4_ does not suit electrochemical applications due to its huge band gap, poor conductivity, and large particle size. To increase the electrochemical biosensing performance of g-C_3_N_4_, researchers have proposed a number of modification techniques to modify the surface of g-C_3_N_4_ [41,42].

For biosensing purposes, g-C_3_N_4_ is a highly potent electrode fabrication material. Furthermore, the physically/chemically modified g-C_3_N_4_-based biosensors have many advantages due to their biocompatibility, low production costs, lack of toxicity, high surface area/volume ratio, and environmentally friendly synthesis process. Reasonably low costs are achieved due to the ease with which bulk g-C_3_N_4_ can be produced via thermal condensation of minimal cost nitrogen-rich precursors, including urea, dicyandiamide, melamine, cyanamide, and others. To tailor the biosensor to a specific set of parameters, g-C_3_N_4_ can be functionalized or element-doped. It has been demonstrated that the result of the constructed sensor is significantly influenced by the interaction of the g-C_3_N_4_ layer with the conductive substrate on the surface of the electrode [43,44,45,46]. Nevertheless, for the production of highly sensitive biosensors, a large-area, homogeneous, and undamaged g-C_3_N_4_ film is preferable.

**Figure 3:**
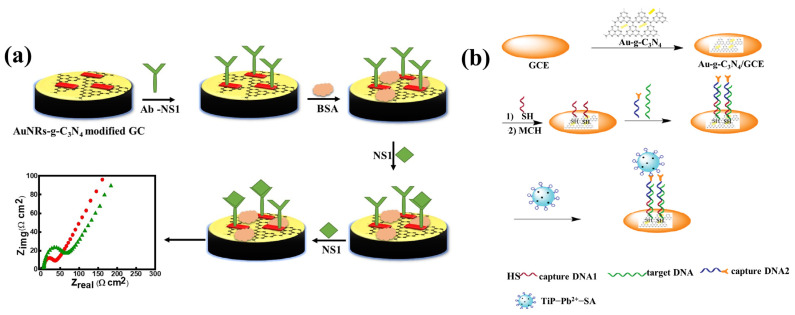
(**a**) Fabrication of NS1 immunosensing platform by using graphitic carbon nitride and gold nanorods; reproduced with permission from [47]. (**b**) Stepwise construction of immunosensing platform utilizing gold nanorods and graphitic carbon nitride for detection of chronic lymphocytic leukemia. Reproduced with permission from [49].

Recently, Ojha et al. [47] constructed a high-throughput NS1 immunosensor for dengue detection, which was constructed by fabricating the working electrode surface of the glassy carbon electrode (GCE) with gold nanorods-adorned graphitic carbon nitride (AuNRs-g-C_3_N_4_) as shown in Figure 3a. First, an NS1 antibody was anchored to the modified GCE as an impedimetric sensing probe for the electrochemical impedance spectroscopy-based detection of NS1 antigen. [Fe (CN)_6_]^3−/4−^, which is the redox pair, was used to track the equivalent changes in charge-transfer resistance (Rct) brought on by an antigen–antibody interaction in PBS buffer as well as in a human serum sample.

In another report, Nirbhaya et al. [48] have devised an ultra-sensing platform for the detection of food toxin (Aflatoxin B1, AfB1) based on thionine-functionalized g-C_3_N_4_. For this purpose, the researchers have polycondensed the melamine and then chemically exfoliated the resultant monolayer to obtain g-C_3_N_4_. In the second step, g-C_3_N_4_ (Thn/g-C_3_N_4_) was functionalized with thionine and electrophoretically deposited onto a glass electrode with an indium tin oxide (ITO) coating. In the last step, anti-aflatoxin B1 (anti-AfB1) was immobilized on the surface of (Thn-g-C_3_N_4_/ITO) via EDC-NHS chemistry, and BSA molecules were added to block non-specific sites.

Afzali et al. [49] have reported the chronic lymphocytic leukemia biosensor, which was constructed by using g-C_3_N_4_ and gold nanorod composite (AuNR/g-C_3_N_4_). The stepwise construction of the nanobiosensor has been illustrated in Figure 3b. On the basis of the increased electrocatalytic activity and electron transport of protonated g-C_3_N_4_ nanosheet (NSs) composites doped with spongy cylindrical polypyrrole (CSPPy-g-CNH NSs), Shrestha et al. have constructed a novel enzyme-based electrochemical biosensor for detection of cholesterol [50]. For construction of the biosensing platform, cholesterol oxidase (ChOx) was immobilized at physiological pH before being used to create nanohybrid composites as prepared. The CSPPy-g-C_3_N_4_H^+^ composite’s large specific surface area and positive charge have the propensity to produce strong electrostatic interaction with ChOx’s negative charge, resulting in the formation of a stable bio-nanohybrid composite with a significant enzyme loading. In an electrochemical detection system, g-C_3_N_4_ has been employed as a synthetic enzyme signal amplification tag due to its peroxidase-like activity. Its utility in electrochemical biosensors is further established by the catalytic reaction brought on by peroxidase mimicking g-C_3_N_4_, which significantly improves voltametric and amperometric signals. A “signal on” immuno-sensor with g-C_3_N_4_-Pd NPs for the robust and specific detection of saxitoxin (STX) was reported based on this technology [51]. An additional study found that in Tris-HCl buffer (pH 7), the positively charged STX and the negatively charged citrate ion-modified g-C_3_N_4_/Pd NPs engaged in improved electrostatic interactions, which led to the adsorption of the nanozyme on the as-formed STX-antibody sandwich immunocomplex. As a result, there was a direct linear association between the concentration of STX and the rate of TMB oxidation. In another study, Zhu et al. have designed an inexpensive, label-free aptasensor for ochratoxin. A non-covalent interaction between g-C_3_N_4_ NSs and free ssDNA on an electrode surface has been used to construct a biosensing system for target identification [52]. To produce a significant current response, the collected g-C_3_N_4_ NSs in turn stimulated the oxidation of H_2_O_2_. This approach differs from others in that it does not call for the immobilization of g-C_3_N_4_ NSs beforehand or any sophisticated labelling approaches. In the composite, amino functionalized g-C_3_N_4_ NSs were used for interaction with the aptamer and to speed up the capture of Cd^2+^ for its quick testing. The summary of the discussed graphitic carbon nitride-based nanobiosensors with other electroanalytical parameters is given in Table 1.

### 1.4. Graphene-Based Nanobiosensors

The 2D nanomaterial graphene has drawn considerable and great interest among researchers due to its distinct features. The most popularly used nanomaterials for making nanobiosensors are derivatives of graphene, including graphene oxide (GO) and reduced graphene oxide (rGO), because of their biocompatibilities. The groups suitable for the electrode surface functionalization using graphene-based materials are abundantly found to be oxygen based. The large surface area to volume ratio enables such nanomaterials to improve biosensing by increasing the immobilization of bio-receptors [53]. These materials are being used in an increasing number of research articles, and the majority of these articles are applicable in biomedical fields, such as drug-delivery, tissue engineering, biosensing, stem cell, protein and nucleic acid-based studies, toxicity research, and imaging [54]. Graphene is comprised of carbon atoms organized in an atomically thin honeycomb lattice. The Scotch-tape approach, which was initially used to isolate graphene from a highly orientated pyrolytic graphite, yields atomically stable graphene at room temperature [55,56,57,58,59,60]. The graphene material itself can be stacked to form three-dimensional layers, wrapped into a zero-dimensional fullerene, or coiled into a one-dimensional carbon nanotube. The electrical spectrum, mechanical, optical, thermal, and electrochemical properties of graphene are just a few significant features [61].

The paper-based electrodes modified with graphene can be used to make flexible, disposable, and light electrochemical sensor systems, and they have an ease of manufacturing processes and lower production costs [62]. A direct writing technique employing a ballpoint pen and specially formulated conductive ink serves to illustrate how much simpler its manufacture is than that of other kinds of electrodes. The paper-based electrodes have proven to be highly selective when it comes to detecting hydrogen peroxide in wastewater [63]. Additionally, it has shown that it is capable of the concurrent detection of cancer biomarkers, such as carcinoembryonic antigen (CEA) and alpha-fetoprotein (AFP), in actual human serum [61].

Additionally, Tuteja et al. [62] designed an immunosensor with a simple procedure by electrochemically functionalizing graphene with 2-aminobenzyl amine (2-ABA) for cardiac troponin I (cTnI) detection. The researchers employed drop casting to add graphene to the gold IDE after it has been constructed on a silicon substrate. The electrochemical modification of the graphene with 2-ABA came next and then the anti-cTnI immobilization procedure at room temperature, which took around 3 h to complete. The I-V output of the immunosensor was observed to have a greater linear range for antigen detection (0.01 to 1 ng/mL), with a limit of detection of 10 ng/mL. Actual human serum samples were further taken and discovered that, with a reaction time of just ten minutes, it worked almost as well as synthetic antigen in buffer media.

**Figure 4:**
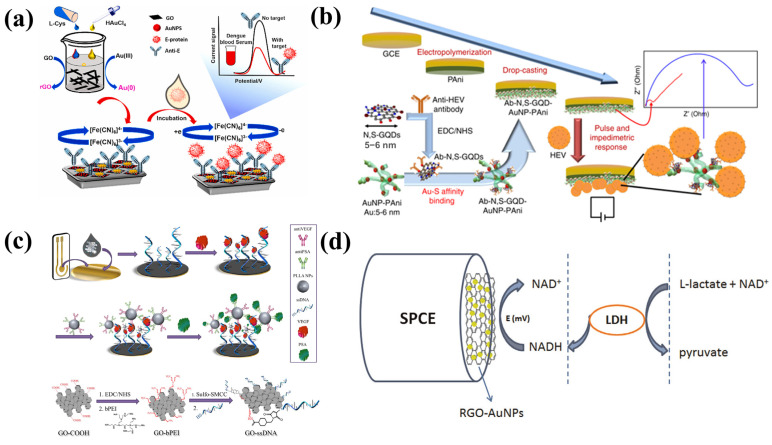
(**a**) An electrochemical immunosensor platform was created for the detection of dengue virus type E-proteins using in situ reduced and modified gold nanoparticle-adorned heteroatom-doped reduced graphene oxides nanocomposites (DENV–E protein). Reproduced with permission from [63]. (**b**) Schematic illustration of the pulse-induced impedimetric sensing of HEV using the Ab-N,S-GQDs@AuNP-PAni nanocomposite-loaded sensor electrode. Reproduced with permission from [64]. (**c**) Process flow diagram for creating GO-ssDNA. With permission, reproduced from [65]. (**d**) Schematic illustration of the LDH/rGO-AuNPs/operation, SPCE biosensors. Reprinted with permission from [66].

Figure 4a shows how functionalized gold nanoparticles-adorned heteroatom-doped reduced graphene oxide nanocomposites (AuNPs/NSG) were employed by Sangili et al. to build an efficient label-free electrochemical immunosensor platform [63]. In this, synthesis method, L-cystein (L-cys) was employed as a reducing and stabilizing agent for Au(III) and graphene oxide. This sensing platform is appropriate for immobilizing more antibodies. Unusually, the extremely crystalline AuNP was grafted onto a layer of 2D graphene. Under ideal situations, the as-built immunosensor resulted in showing a broad linear operating range of 0.01 to 100 ng/mL and a low limit of detection of 1.6 pg/mL for DENV-E detection. The developed sensor exhibits excellent selectivity for identifying DENV-E against their antibodies, including DENV that is closely related to it.

Recently, Chowdhury et al. have designed an electrochemical biosensor for hepatitis E virus detection (Figure 4b) [64]. This pulse-triggered ultrasensitive sensor was created with polyaniline nanowires and graphene quantum dots and embedded gold by using an interfacial polymerization and subsequent self-assembly method. For the diagnosis of prostate cancer (PCa) at an early stage, a straightforward and accurate tool must be created due to the increased surface of the viral particle and the length of the antibody-conjugated polyaniline chain in comparison to the existing methods. They have described a unique fabrication technique for a dual-modality biosensor to concurrently detect PSA and VEGF in human serum. The reported device was created by fabricating the Au electrode with graphene oxide/ssDNA (GO-ssDNA) for VEGF detection. Poly-L-lactide nanoparticles (PLLA NPs) were also added for signal enhancement as well as for PSA detection. The results indicated that the developed biosensor has broad linear detection ranges of 0.05 to 100 ng/mL for VEGF and 1 to 100 ng/mL for PSA. The electrochemical sensors improve the sensitivity toward HEV when an external electrical pulse is applied during the virus accumulation process. The sensor was used to assess a range of HEV genotypes, including G1, G3, G7, ferret HEV, which was isolated from a cell culture supernatant, and many fecal specimen samples that were collected from a monkey that was infected with G7 HEV. The sensitivity can be identified similarly to real-time quantitative reverse transcription-polymerase chain (RT-qPCR). The findings indicate that the suggested biosensor has the potential to enable the evolution of dependable, high-performance sensing approaches for HEV detection [64].

Figure 4c shows the construction of a biosensor developed for the early prostate cancer diagnosis. In order to diagnose prostate cancer (PCa) as early as feasible, prevent metastases, and start treatment as soon as possible, Pan et al. have presented a novel fabrication approach for a dual-modality biosensor [65]. This biosensor can simultaneously detect PSA and VEGF in human serum. For VEGF detection, an Au electrode was coated with graphene oxide/ssDNA (GO-ssDNA), and poly-L-lactide nanoparticles (PLLA NPs) were added for signal enhancement and PSA detection. The constructed biosensor was able to efficiently detect prostate cancer and show broad linear detectable ranges of 0.05 to 100 ng/mL and 1 to 100 ng/mL for VEGF and PSA, respectively.

To detect l-lactate, Azzouzi et al. designed a unique amperometric biosensor. To achieve this, they synthesized the sensing probe by adorning reduced graphene oxide with l-lactate dehydrogenase (LDH) and gold nanoparticles (rGO-AuNPs) (Figure 4d) [66]. First, tests for NADH detection using screen-printed electrdes modified with rGO-AuNPs revealed a dynamic calibration range and very low detection limit. A sol–gel matrix made of methyltrimethoxysilane and tetramethoxysilane was then used to assemble the biosensor, which included both the enzyme and rGO-AuNPs. There was optimum coenzyme concentration, functioning pH, and enzyme loading. With an excellent specific sensitivity of 154 µA/mM cm^2^ and a detection limit of 0.13 µM, the sensor responded linearly to l-lactate in the concentration range of 10 µM to 5 mM.

**Figure 5:**
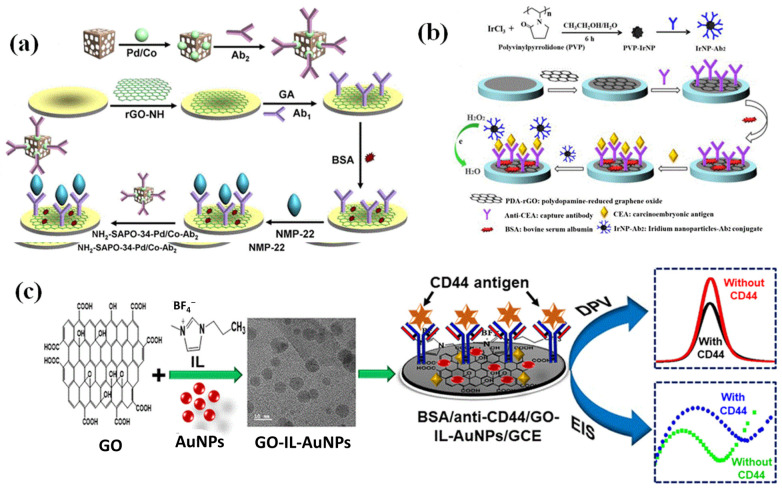
(**a**) A schematic diagram showing the fabrication of the NH_2_-SAPO-34-Pd/Co-Ab2 and the step-by-step construction of modified immunosensor. (**b**) The steps involved in making an electrochemical immunosensor for measuring carcinoembryonic antigen. (**c**) Fabrication of an electrochemical immunosensor to measure and identify the very sensitive breast cancer biomarker CD44 (cluster of differentiation-44). Reproduced with permission from [67,68,69].

In order to detect the cancer biomarker nuclear matrix protein-22 (NMP-22) present in the bladder, Wu et al. have created a novel sandwich-type electrochemical immunosensor (Figure 5a) that uses labels called NH_2_-SAPO-34-supported Pd/Co nanoparticles (NH_2_-SAPO-34-Pd/Co NPs). Immobilized primary antibodies were utilized due to rGO-high NH’s conductivity and vast surface area (Ab1) [67]. NH_2_-SAPO-34-Pd/Co NPs was utilized as labels, and a secondary antibody (Ab2) was immobilized by means of the Pd/Co NPs’ adsorption capacity to protein because of their exceptional catalytic activity toward hydrogen peroxide. The designed immunosensor had a relatively low limit of detection 0.33 pg/mL and a large linear calibration range of 0.001 to 20 ng/mL. The examination of clinical urine samples has demonstrated satisfactory reproducibility and stability. This innovative and highly sensitive immunosensor shows promise for use in the detection of many tumor markers.

In order to accurately measure a carcinoembryonic antigen, Miao et al. reported a sandwich-style electrochemical immunoassay (CEA). To boost the strength of the electrical signal, iridium nanoparticles (Ir NPs) were applied onto the GCE surface [68]. For the construction of an electrochemical immunoassay, the main antibody (Ab1) against CEA was first immobilized on polydopamine-reduced graphene oxide (PDA-rGO), as depicted in Figure 5b. As a second step, they have immobilized secondary antibodies (Ab2) onto the Ir-NPs to use as signal markers. This sensitive test for CEA is the consequence of the combination of the high surface area of PDA-rGO with the outstanding electro-oxidative H_2_O_2_-sensing characteristics of IrNPs. The optimal operating voltage for the assay was 0.6 V (vs. SCE), which allowed for a linear range of 0.5 pg/mL to 5 ng/mL and a 0.23 pg/mL detection limit. The immunosensor’s repeatability and consistency prove it to be a solid immunoassay method for detecting tumor biomarkers. It was used to analyze serum samples with added CEA.

Recently, Ranjan et al., as depicted in Figure 5c, reported the development of a an electrochemical immunosensor for the highly sensitive detection and quantification of the cluster of the differentiation-44 (CD44) antigen, which is a known biomarker for breast cancer [69]. Immobilized on a GCE, the hybrid composite is comprised of graphene oxide, ionic liquid, and gold nanoparticles (GO-IL-AuNPs). Because of the abundance of oxygen functions, GO is ideally suited for the immobilization of antibodies. However, the immunosensor’s functionality is improved by the combination of 1-butyl-3-methylimidazolium tetrafluoroborate (BMIM.BF4) with AuNPs, which improves electron transport and boosts the immunosensor’s effective surface area. Differential pulse voltammetry and EIS detection methods have been used to successfully quantify CD44 antigen. The proposed immunosensor has been tested, and the results show that it performs admirably in both phosphate-buffered saline (PBS) and serum samples. In PBS, the immunosensor has a limit of detection of 2.0 fg/mL as determined by DPV and 1.90 fg/mL as determined by EIS, within a linear range of 5.0 fg/mL to 50.0 µg/mL. The immunosensor’s great sensitivity and selectivity make it a useful tool for detecting the CD44 antigen in clinical specimens.

**Figure 6:**
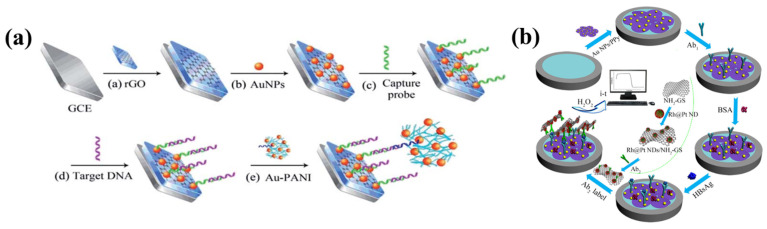
(**a**) A schematic depiction of the stepwise electrode modification process and detection of target DN. (**b**) A flowchart illustrating the stepwise processes involved in the fabricating of a sandwich-type electrochemical immunosensor for the detection of hepatitis B surface antigen. Reproduced with permission from [70,71].

Figure 6a shows a novel ultrasensitive DNA biosensor reported by Liu et al. for M. tuberculosis-specific DNA insertion sequence IS6110 detection. The biosensor is based on a sensing platform comprised of reduced graphene oxide–gold nanoparticles (rGO-AuNPs) and a tracer label composed of gold nanoparticles–polyaniline (Au-PANI) for amplification [70]. In this case, due of its high surface area and biocompatibility, reduced graphene oxide was utilized as the sensor matrix. AuNPs were further electrodeposited onto the modified electrode surface, which improved the capture probe’s immobilization and facilitated electron transfer. Excellent electrochemical activity and good biocompatibility were displayed by the Au-PANI nanocomposite. In order to provide a quick setup procedure for a signal-on DNA biosensor, it was utilized as a tracer label for electrochemical detection. The Au-PANI nanocomposite’s strong electroactivity allowed for the creation of a DNA biosensor with a high sensitivity for the quantification and detection of M. tuberculosis over a very large linear calibration range of 1.0 × 10^−15^ to 1.0 × 10^−9^ M.

A sandwich-type electrochemical immunosensor was constructed by Pei et al. to detect the hepatitis B surface antigen quantitatively (HBsAg) [71]. Gold nanoparticles decorated on polypyrrole nanosheets served as the platform for the immunosensor, while Rh core and Pt shell nanodendrites decorated onto amino group-modified graphene nanosheets (RhPt NDs/NH_2_-GS) served as the labels (Figure 6b). An increase in the immunosensor’s current signal was seen when RhPt NDs/NH_2_-GS was used as the label. Due of their branched core-shell structure, RhPt NDs contain a large number of catalytic active sites. In addition, Au NPs/PPy NS improved electron transport and offered a favorable milieu for successfully immobilizing antibodies and increasing the sensitivity of the immunosensor. In addition to these advantages, the immunosensor demonstrated excellent stability, selectivity, and repeatability across a range of concentrations from 0.0005 to 10 ng/mL, a 166 fg/mL limit of detection for HBsAg (S/N = 3), and a linear relationship between these parameters. Further, the immunosensor showed promise in clinical analytical applications due to its robust accuracy when testing against genuine serum samples.

**Figure 7:**
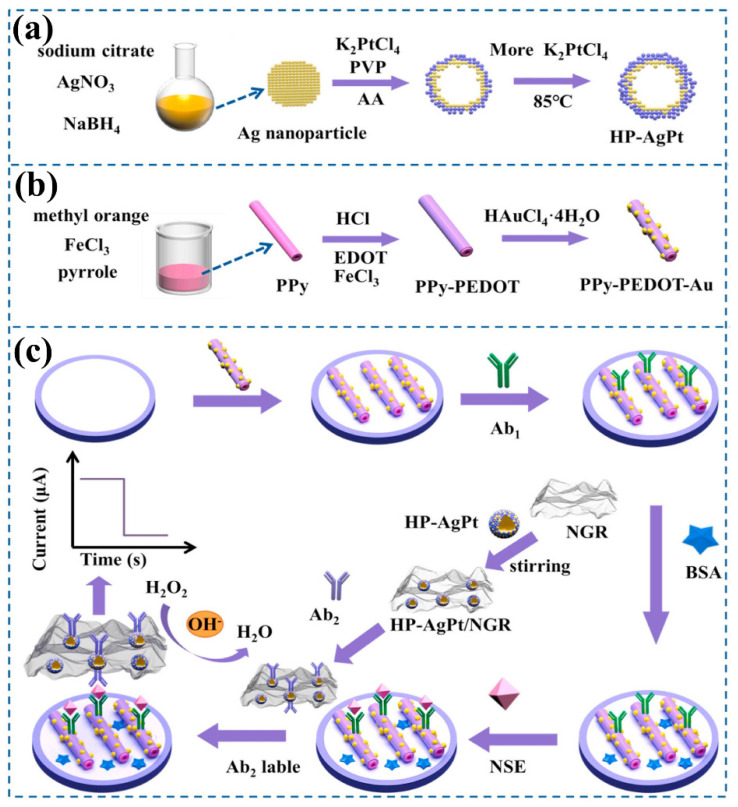
The schematic pathway for the synthesis for HP-Ag/Pt (**a**) and PPy-PEDOT-Au (**b**) and the steps involved in immunosensor fabrication (**c**). Reproduced with permission from [72].

Recent studies by Tang et al. presented an electrochemical immunosensor employing a two-fold signal enhancement and a sensitive approach to detect neuron-specific enolase [72]. In order to design a dual signal amplifier, they functionalized the N-atom-doped graphene using hollow porous Pt-skin Ag-Pt alloy (HP-Ag/Pt/NGR) as shown in Figure 7. This structure enables greater atomic utilization as well as a large increase in the number of electroactive centers, with a remarkable electrocatalytic activity and endurance for H_2_O_2_ reduction. To double amplify the current signal, NGR with high catalytic activity is employed as a support material for HP-Ag/Pt.

Sainz and his coworkers have developed a lactate determination electrochemical biosensor [73]. The enzyme lactate oxidase (LOx) has been connected to chevron-shaped graphene nanoribbons (GNR), which were previously created using a solution-based chemical process and used as modifier to glassy carbon electrodes in order to fabricate a biosensor. Initially, the researchers electrochemically reduced the matching 4-carboxyphenyl diazonium salt to graft a 4-carboxyphenyl layer onto the GNR-modified electrode surface. Covalent immobilization of the enzyme is achieved through the formation of an amide bond between its carboxylic and amine groups, which is made possible by the exposure of the carboxylic groups to the solution.

**Figure 8:**
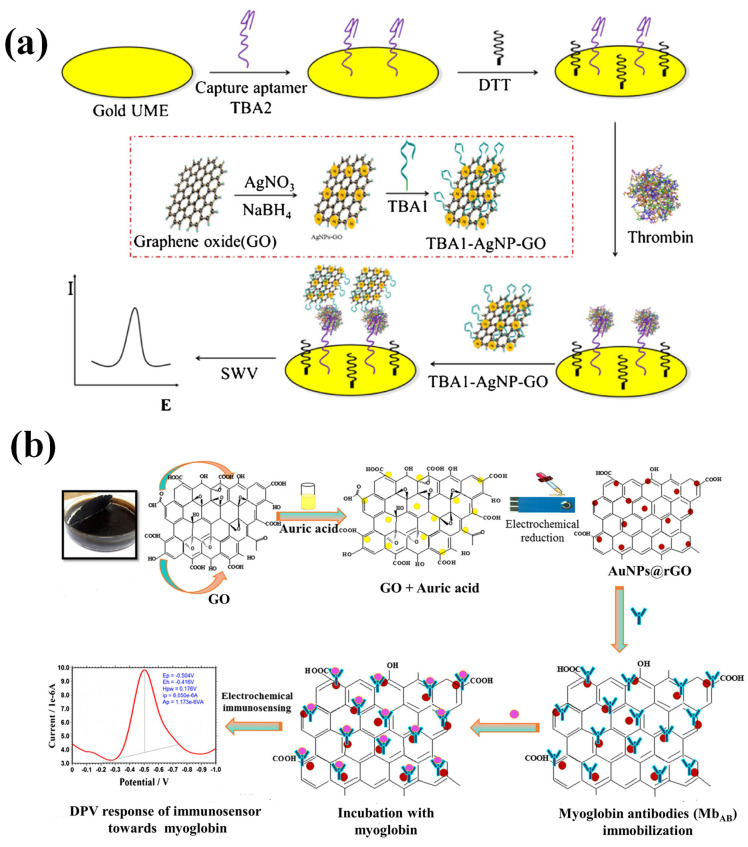
(**a**) Pictorial representation of an electrochemical aptasensor for thrombin using gold UME. The TBA1-AgNP-GO complex serves as the signal probe. (**b**) A schematic illustrating the stepwise processes for fabricating an AuNPs@rGO composite-based myoglobin immunosensor. Reproduced with permission from [74,75].

To detect thrombin, scientists created an electrochemical aptasensor with high sensitivity. Graphene oxide (GO) coated with silver nanoparticles (AgNP) were positioned on a microelectrode and are the basis of this method, which amplifies the signal [74]. Chemical reduction was used to produce silver nanoparticle-decorated graphene oxide (AgNP-GO), which was then chemically functionalized with a thrombin binding aptamer 1 (TBA1; a 29-mer) bearing a 3′-terminal thiol group. For the purpose of creating a signaling probe (Figure 8a), it was adsorbed on the AgNP-GO using a self-assembled monolayer approach (TBA1-AgNP-GO). Gold electrode surface was self-assembled with a capture thrombin-binding aptamer 2 (TBA-2; a 15-mer) that has a 3′-terminal thiol group. TBA2-thrombin-TBA1-AgNP-GO sandwiches are generated after thrombin binds to the signaling probe. Then, the AgNP voltametric signal, a square wave, is captured. The limit of detection for thrombin was 0.03 nmol/L (at S/N = 3), and the oxidation signal (measured at 0.21 V vs. Ag/AgCl) rises in a linear manner from 0.05 to 5 nmol/L. The aptasensor detects only its target protein and is unaffected by any others. Further, it was used for the effective analysis of thrombin in human serum that had been adulterated.

To employ myoglobin as a biomarker of heart health, an electrochemically produced composite of gold nanoparticles and reduced graphene oxide (AuNPs@rGO) on a screen-printed electrode (SPE)S was created [75]. As can be seen in Figure 8b, an antibody against cardiac myoglobin was manufactured in-house and then immobilized on the surface of an electrode to construct the immunosensor. In order to monitor the immunosensing response, differential pulse voltammetry was performed, and the results showed a reduction peak at about 0.5 V (vs. Ag/AgCl). The appearance of the reduction peak is generated by the reduction in the iron moiety in the myoglobin’s heme group. The detection limit of the immunosensor for cardiac myoglobin was found to be 0.67 ng/mL and with a wide linearity range of 1 ng/mL to 1400 ng/mL. In terms of detection limit, the results obtained were about eight times better than those obtained using ELISA testing (with a limit of detection of 4 ng/mL) using the similar antibodies. Additionally, samples of spiked serum were examined using the immunosensor.

According to a different study, a platform for sensing multi-cardiac biomarkers was developed employing aptamer-based electrochemical sensors for cardiac troponin I (cTnI) and brain natriuretic peptide (BNP-32) [76]. Here, electrophoretically produced polyethyleneimine/reduced graphene oxide sheets were used in conjunction with commercially available gold-based screen-printed electrodes. By incorporating propargyl groups onto the electrode via covalent grafting of propargylacetic acid, the azide-terminated aptamers can be immobilized using Cu(I)-based “click” chemistry. By adding poly(ethylene glycol) units to pyrene anchors, cardiac sensors were able to achieve both low biofouling and good selectivity. The developed BNP-32 biosensor displayed a dynamic linear range of 1 pg/mL to 1 µg/mL in blood, and the cTnI biosensor exhibits a linear response of 1 pg/mL to 10 ng/mL, both of which are required for the initial phase analysis and diagnosis of cardiac failure. Major progress toward multianalyte sensing of cardiac biomarkers has been made with the creation of such electrochemical aptasensors. The summary of the graphene-based nanobiosensors with electroanalytical parameters is tabulated in Table 2.

### 1.5. Black Phosphorous-Based Nanobiosensor

**Figure 9:**
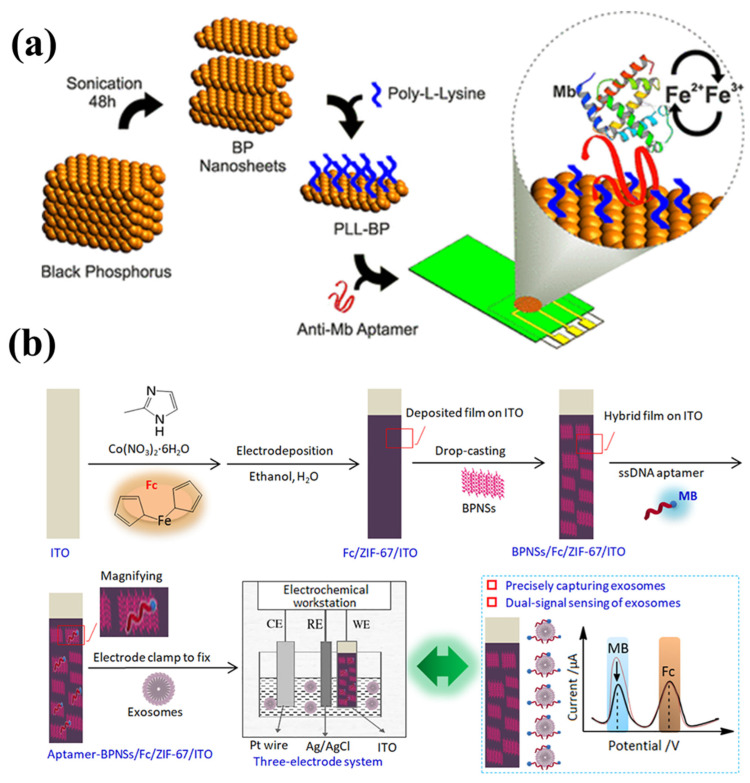
(**a**) An illustration of the steps involved in creating a bio interface on an electrode for Mb detection, including liquid phase exfoliation of BP nanosheets and surface modification of those nanosheets. (**b**) Schematic of fabrication, characterization, and application of the aptasensor of exosomes for dual-signal and intrinsic self-calibration using a functional hybrid thin-film sensing platform aptamer-BPNSs/Fc/ZIF-67/ITO. Reproduced with permission from [88,89].

Black phosphorus (BP) is a kind of phosphorus that has recently gained interest as a promising biosensing material due to its status as a promising second-generation two-dimensional (2D) material [77,78,79]. The increased charge carrier mobility (about 1000 cm^2^ V^−1^ s^−1^) and thickness-dependent bandgap (0.3 to 2.0 eV) have led to its usage in gas sensors and field effect transistors [80,81,82,83,84,85,86,87]. It can be functionalized covalently as well as non-covalently, which makes it as promising material as an electrode modifier for biosensing applications. Using direct electron transfer and aptamer-functionalized black phosphorus nanostructured electrodes, Kumar et al. recently showed that the redox-active cardiac biomarker myoglobin (Mb) may be electrochemically identified [82] as shown in Figure 9a. Furthermore, they have graphed poly-L-lysine (PLL) onto the few-layer black phosphorus nanosheets synthesized in order to improve its interaction with anti-Mb DNA aptamers. They then tested the aptasensor using Mb-containing serum samples and discovered that it exhibits a detection limit of 0.524 pg/mL with a robust linear response of 1 pg/mL to 16 µg/mL. This technique opens a path for multiplexed point-of-care diagnostics of cardiovascular disorders in challenging human samples.

Similarly, Sun et al. have engineered a unique hybrid thin-film technology to design an aptasensor for exosomes with dual signals and inherent self-calibration [89]. Integration of a methylene blue (MB)-labeled single-strand DNA aptamer on an indium tin oxide (ITO) slice was the first step in the construction of this platform (Figure 9b). Next, black phosphorous nanosheets (BPNSs) and ferrocene (Fc)-doped metal–organic frameworks (ZIF-67) were assembled on the ITO. The aptamer-BPNSs/Fc/ZIF-67/ITO-sensing platform that resulted was capable of achieving dual redox-signal responses of MB (labeled on aptamer) and Fc (labeled on BPNSs) (doped into ZIF-67). Even in the presence of some cancer cell-derived exosomes, the redox current of MB was consistently reduced, whereas the redox current of Fc (as a control) was minimally affected. The detection limit for exosomes was shown to be as low as 100 particles/mL, demonstrating the success of developing an intrinsic self-calibration aptasensor. Additionally, they have tested their newly designed aptasensor on actual human plasma and blood samples from healthy people as well as breast cancer patients. The aptasensor’s capacity for precise protein capture enables rapid identification of particular exosomes produced by cancer cells. The ability of this aptasensor to identify a wide range of biomarkers in exosomes extracted from cell lines makes it a valuable tool for researching cutting-edge approaches for a highly efficient analysis of exosomes generated from diverse cancer cells.

Ramalingam et al. reported an electrochemical microfluidic biochip for the detection of okadaic acid (OA). For the construction of a sensing probe, they have modified the screen-printed carbon electrodes (SPCEs) by immobilizing an aptamer specific for OA onto a phosphorene–gold nanocomposite [90]. Without the need for a reducing agent, BP–Au nanocomposites were produced in situ, on-step. Signal strength was measured using potassium ferro-ferricyanide as a redox pair. A microfluidic platform was built with the goals of speeding up reactions, raising sensitivity, and making the system more portable. Various functions, such as sample mixing and incubation, were implemented in this apparatus by use of channels designated for those tasks. As a whole, the OA detection system was comprised of a polydimethylsiloxane microfluidic chip that contained an aptamer-modified SPCE. Spectroscopic and electrochemical analysis was performed on the produced nanomaterials and microfluidic channels. Based on differential pulse voltammograms, a wide linear range for this assay was found from 10 nM to 250 nM, with a 25 pM detection limit. Research on species selectivity was also conducted using spiked mussel samples and other potential contaminants. They have reported this point-of-care gadget can be used in fishing units for on-the-spot testing. The summary of the discussedblack phosphorous-based nanobiosensors with electroanalytical parameters have been given in Table 3.

### 1.6. MXenes-Based Nanobiosensors

Two-dimensional inorganic substances called MXenes are extremely thin (only a few atomic layers thick). Because of their composition as nitrides, carbonitrides, or transition metal carbides, examples including titanium carbonitride (Ti_2_CN) and titanium carbide (Ti_3_C_2_), they exhibit remarkable qualities, such as excellent conductivity, outstanding fluorescence, and plasmonic and optical characteristics [91,92,93]. They have also been used as an electrode modifier for the modification of various types of electrodes used for biosensing applications [94]. The electrochemical MXene/GCPE sensor was created to find adrenaline.

Recently, Wang et al. have described a label-free electrochemical immunosensor that uses paper and has a working electrode modified with MXene (Ti_3_C_2_) nanosheets for the detection of cTnI. To immobilize the bio-receptor (anti-cTnI), the MXene nanosheets were functionalized with aminosilane and then adsorbed onto the working electrode with Nafion [95]. The large surface area and high conductivity of MXene nanosheets facilitate the immobilization of the bio-receptor and the transfer of electrons between electrochemical species and the underlying electrode. Thus, the created immunosensor could detect cTnI at a wide linear range from 5 to 100 ng/mL, with a detection limit of 0.58 ng/mL. Additionally, the immunosensor displayed high levels of selectivity and reproducibility. The electrochemical immunosensor enabled a sensitive and quick detection of cTnI, suggesting its probable application in a cost-effective, real-time monitoring of AMI cases in hospitals.

**Figure 10:**
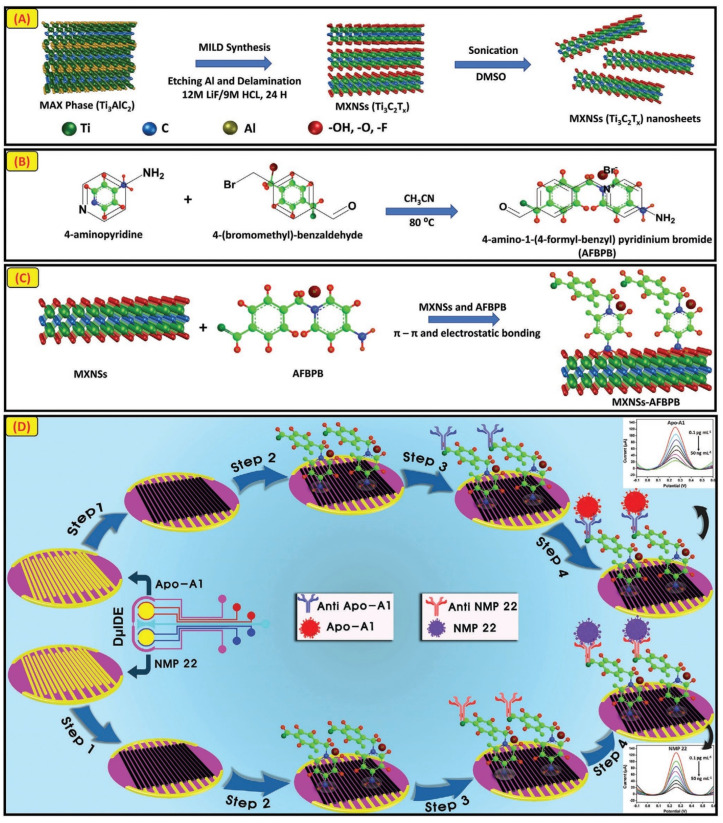
(**A**) Conceptual illustration of the synthesis of MXNSs-Ti_3_C_2_Tx. (**B**) A diagram illustrating the production of the multipurpose 4-amino-1-(4-formylbenzyl) pyridinium bromide (AFBPB) TSIL. (**C**) AFBPB is used in the MXNS functionalization as shown in the schematic. (**D**) A diagram outlining the procedures for developing the immunosensor for detecting NMP 22 and Apo-A1. Reproduced with permission from [96].

In a different study, Sharifuzzaman et al. revealed the electroMXenition method, a one-pot, environmentally friendly electroplating technique that can quickly fabricate the conductive electrodes with 2D MXene-Ti_3_C_2_Tx nanosheets (MXNSs) (Figure 10) [96]. An electric field is produced as a result of the redox process occurring in the colloidal MXNS solution while being governed by a constant applied voltage. This electric field cathodically electroplates the nanoparticles. In order to significantly immobilize MXNSs and covalently bind antibodies, a multiplex host arena made of 4-amino-1-(4-formyl-benzyl) pyridinium bromide (AFBPB) was used. Exploring the advantages of AFBPB coated on MXNSs led to the microfabrication of a single-masked, dual interdigitated gold microelectrode (DIDE). The DIDE biosensor modified with the MXNSs-AFBPB-film displayed a seven times higher redox current than bare electrodes. The novel designed dual immunosensor displayed precise and broad linear ranges over five orders of magnitude when model bladder cancer analytes Apo-A1 and NMP 22 were employed. The limit of detection for these analytes was found to be 0.3 and 0.7 pg/mL, respectively. The summary of MXenes-based nanobiosensors with electroanalytical parameters and a real sample chosen for the study, etc., have been given in Table 4.

## 2. Conclusions and Future Prospective

We have discussed the nanobiosensors based on two-dimensional (2D) materials, mainly graphitic carbon nitride (g-C_3_N_4_), graphene oxide, black phosphorous, and MXenes, which has been utilized for the diagnosis of various infectious diseases at initial stages. This review article will be quite useful for the researchers working in the field of nanobiosensors design for infectious disease. Nanotechnology has the potential to enhance the performance of nanobiosensors, which are sensitive to approaches that are especially useful for the early identification of diseases, if integrated into sensor systems and put to use in this area. Nanomaterials, including metallic nanoparticles, carbon-based nanomaterials, polymeric nanostructures, magnetic nanomaterials, quantum dots, nanowires, or nanomembrane structures, are the building blocks of nanobiosensors, which provide these systems a substantial advantage in biosensing. In addition to their high sensitivity, the huge surface-to-volume ratios and good conductivity of these materials make them ideal candidates for the designs of nanobiosensors. It is undeniable that the extreme sensitivity, high specificity, and selectivity afforded by the affinity between a bioreceptor molecule (such as an enzyme, peptide, antibody, or aptamer) and a target analyte (such as a protein, biomarker, or gene) is the result of this affinity. Nanobiosensors are fast, highly sensitive, as well as selective analytical tools for the diagnosis of several diseases, in contrast to conventional methods, such as chromatographic and spectroscopic methods, which take a long time, use a lot of organic solvents, and involve the usage of harmful chemicals. Nanobiosensors are a fantastic analytical instrument for diagnosing diseases because of their many useful characteristics, including their precision, reproducibility, dynamic capacity change, and sensitivity to environmental changes, such as pressure, pH, and temperature. Even yet, more people need to be made aware of the potential of nanobiosensors in the business world. Several methods exist for incorporating nanobiosensors into future smart gadgets and systems that can be operated from a distance. Biochips that are both cost-effective and versatile can be used to detect multiple biomarkers at once. In addition, micromotors and microconsoles, which are examples of self-propelled sensors, can play a role in expanding the usefulness of nanobiosensors in this new context.

## Figures and Tables

**Table 1:** Summary of discussed graphitic carbon nitride-based nanobiosensors with electroanalytical parameters.

Nanobisensor	Techniques	Analyte	Linear Calibration Range	Limit of Detection	Real Sample	Reference
GC/g-C_3_N_4_	DPV	Dopamine	10^−8^–10^−6^ M	10^−8^ M	-	[40]
NiS/S-g-C_3_N_4_	LSV, AMP	Glucose	1–2100 µM	1.5 µM	-	[46]
AuNRs-g-C_3_N_4_	IMP	NS1	0.6–216 ng mL^−1^	0.09 ng mL^−1^	Blood Serum	[47]
Thn/g-C_3_N_4_/ITO	DPV	Aflatoxin B1	1 fg mL^−1^–1 ng mL^−1^	0.328 fg mL^−1^	Human Serum	[48]
Au nanorods/g-C_3_N_4_	DPV	Target DNA	0.6–6.4 nM	20 pM	-	[49]
ChOx-CSPPy-g-C_3_N_4_H^+^	AMP	Cholesterol	0.02–5.0 mM	8.0 μM	Human Serum	[50]
g-C_3_N_4_-Pd NPs	CA	Saxitoxin	20–400 pg mL^−1^	1.2 pg mL^−1^	Seawater and seafood	[51]
g-C_3_N_4_NS	CV	Ochratoxin A	0.2–500 nM	0.073 nM	red wines, juices, corns	[52]

DPV = differential pulse voltammetry, LSV = linear sweep voltammetry, IMP = impedance, CA = chronoamperometry, ChOx = cholesterol oxidase, NS = nanosheet.

**Table 2:** Summary of discussed graphene-based nanobiosensors with electroanalytical parameters.

Nanobisensor	Techniques	Analyte	Linear Calibration Range	Limit of Detection	Real Sample	Reference
f-GN	I-V, LSV	Cardiac Troponin I	0.01–1 ng mL^−1^	0.01 ng mL^−1^	Spiked serum	[62]
AuNPs/NSG	IMP	Dengue virus E-protein	0.01–100 ng mL^−1^	1.6 pg mL^−1^	Blood serum	[63]
N,S-GQDs@AuNP-PAni	CV, IMP	Hepatitis E virus	1 fg mL^−1^–100 pg mL^−1^	0.8 fg mL^−1^	Human serum	[64]
GO/ssDNA/PLLA NPs	DPV	VEGF	0.05–100 ng mL^−1^	50 pg mL^−1^	Human serum	[65]
GO/ssDNA/PLLA NPs	DPV	PSA	1–100 ng mL^−1^	1 ng mL^−1^	Human serum	[65]
rGO-AuNPs	AMP	l-lactate	10 µM–5 mM	0.13 µM	Human serum	[66]
NH_2_-SAPO-34-Pd/Co-Ab_2_	CV	NMP 22	0.001–20 ng mL^−1^	0.33 pg mL^−1^	Urine sample	[67]
PDA-rGO	CV	Carcinoembryonic antigen	0.5 pg mL^−1^–5 ng mL^−1^	0.23 pg mL^−1^	Human Serum	[68]
GO-IL-AuNPs	DPV	CD44 antigen	5.0 fg mL^−1^–50.0 μg mL^−1^	2.0 fg mL^−1^	Human Serum	[69]
GO-IL-AuNPs	IMP	CD44 antigen	5.0 fg mL^−1^–50.0 μg mL^−1^	1.90 fg mL^−1^	Human Serum	[69]
rGO-AuNPs	CV, DPV	M-TB	1.0 × 10^−15^–1.0 × 10^−9^ M	-	-	[70]
Au NPs/PPy NS	AMP	HBsAg	0.0005–10 ng mL^−1^	166 fg mL^−1^	Human Serum	[71]
HP-Ag/Pt/NGR	AMP	NSE	50 fg mL^−1^–100 ng mL^−1^	18.5 fg mL^−1^	Human Serum	[72]
GC/GNR/BzA/LOx	CV	Lactate	3.4 × 10^−5^–2.8 × 10^−4^ M	11 μM	Apple juices	[73]
TBA1-AgNP-GO	SWV	Thrombin	0.05 nM–5 nM	0.03 nM	Human Serum	[74]
AuNPs@rGO	DPV	Myoglobin	1 ng mL^−1^–1400 ng mL^−1^	0.67 ng mL^−1^	Human Serum	[75]
SPE/rGO/aptamer	DPV	BNP-32	1 pg mL^−1^–1 µg mL^−1^	0.9 pg mL^−1^	Human Serum	[76]
SPE/rGO/aptamer	DPV	cTnI	1 pg mL^−1^–10 ng mL^−1^	1 pg mL^−1^	Human Serum	[76]

IMP = impedance, DPV = differential pulse voltammetry, LSV = linear sweep voltammetry, AMP = amperometry, CV = cyclic voltammetry, SWV = square wave voltammetry, NSG = heteroatom-doped reduced graphene oxide, VEFG = vascular endothelial growth factor, PLLA = poly-L-lactide, f-GN = functionalized graphene. HP = hollow porous, GQD = graphene quantum dots, PDA = polydopamine, PSA = prostate-specific antigen, NGR = nitrogen atom-doped graphene, GNR = graphene nanoribbons, BzA = 4-amino benzoic acid, TBA1 = thrombin-binding aptamer 1, BNP-32 = brain natriuretic peptide, cTnI = cardiac troponin I, SPE = screen-printed electrode, rGO = reduced graphene oxide, PSA = prostate-specific antigen, NMP 22 = nuclear matrix protein number 22, NSE = neuron-specific enolase, HBsAg = hepatitis B surface antigen, M-TB = mycobacterium tuberculosis.

**Table 3:** Summary of discussed black phosphorous based nanobiosensors with electroanalytical parameters.

Nanobisensor	Techniques	Analyte	Linear Calibration Range	Limit of Detection	Real Sample	Reference
PLL-BP-Apt	CV	Myoglobin	1 pg mL^−1^–16 μg mL^−1^	0.524 pg mL^–1^	Human Serum	[88]
Aptamer BPNSs/Fc/ZIF-67/ITO	SWV	Exosomes	1.3 × 10^2^–2.6 × 10^5^ particles mL^−1^	100 particles mL^−1^	Human Serum	[89]
SPE-BP-Au	DPV	Okadaic acid	10 nM–250 nM	8 pM	Mussel extract	[90]

DPV = differential pulse voltammetry, AMP = amperometry, CI = capacitance immunosensor, SWV = square wave voltammetry, SPE = screen-printed electrode, PLL = poly-l-lysine, BP = black phosphorus, Apt = aptamer, BPNSs = black phosphorus nanosheets, Fc = ferrocene, ZIF = zeolitic imidazolate framework, ITO = indium tin oxide, PSA = prostate-specific antigen, NMP 22 = nuclear matrix protein number 22.

**Table 4:** Summary of discussed MXenes-based nanobiosensors with electroanalytical parameters.

Nanobisensor	Techniques	Analyte	Linear Calibration Range	Limit of Detection	Real Sample	Reference
Ti_3_C_2_ MXene-NS	CI	PSA	0.1–50 ng mL^−1^	0.031 ng mL^−1^	Human Serum	[92]
Ti_3_C_2_ MXene-NS	DPV	cTnI	5–100 ng mL^−1^	0.58 ng mL^−1^	-	[95]
Ti_3_C_2_Tx-MXene-NS	DPV	NMP 22	0.1 pg mL^−1^–50 ng mL^−1^	0.3 pg mL^−1^	Human Urine	[96]

## Data Availability

Not applicable.
